# Eleventh Annual Report of the Presbyterian Eye, Ear and Throat Hospital

**Published:** 1889-09

**Authors:** 


Eleventh annual report of the Presbyterian Eye, Ear and Throat Charity Hospital. Free dispensary open daily for patients from 1 o'clock to 4, p. m. No. 1007 E. Baltimore St., Baltimore Md. An interesting report kindly sent us by Julian J. Chisolm, M. D., Surgeon-in-Chief of the Hospital. Amongst the numerous cases and operations occurring in this Hospital during the year the one which has attracted most attention was the engrafting of the healthy cornea of a living rabbit to the diseased cornea of a blind man for the purpose of giving him useful sight. The transplanting of the animal graft was successfully performed. It has been retained, and is now a part of the human eye. At first it become cloudy, then commenced slowly to clear up, and now promises to give him the useful sight which he sought by this curious surgical operation. The success of this transplanting operation has brought the Hospital into very prominent notice in all parts of the country.

